# Characteristics of historical precipitation for winter wheat cropping in the semi-arid and semi-humid area

**DOI:** 10.3389/fpls.2023.1049824

**Published:** 2023-04-03

**Authors:** Dan Fang, Jingyao Huang, Weiwei Sun, Najeeb Ullah, Suwen Jin, Youhong Song

**Affiliations:** ^1^ School of Agronomy, Anhui Agricultural University, Hefei, Anhui, China; ^2^ Faculty of Science, Universiti Brunei Darussalam, Gadong, Brunei; ^3^ Anhui Provincial Meteorological Information Center, Anhui, Meteorological Service, Hefei, Anhui, China

**Keywords:** winter wheat, waterlogging, drought, phenological development, precipitation grade, continuous rainfall

## Abstract

Winter wheat (*Triticum aestivum* L.) is one of major crops in the area along Huai river, China where it is a semi-arid and semi-humid region with sufficient precipitation for an entire season, but with uneven distribution within various growth stages. The instability of precipitation is an important factor in limiting wheat production potential under climate change. Therefore, it is essential to characterise the precipitation associated with different crop developmental stages. Based on climate data from 1999 to 2020 in six representative meteorological stations, we characterised the historical precipitation relating to seven key growth stages in winter wheat. There is no clear trend of interannual variation of precipitation for wheat season, with an average of precipitation of 414.4 ± 121.2 mm. In terms of the distribution of precipitation grade within a season, light rain was dominant. Continuous rain occurred frequently during the pre-winter seedling and overwintering stages. The critical period of water demand, such as jointing and booting, has less precipitation. The fluctuation range of precipitation in sowing, heading-filling and maturation stages is large, which means that there is flood and drought at times. In conclusion, these findings provide a foundation for instructing winter wheat cropping in confronting with waterlogging and drought risk due to uneven precipitation in ‘Yanhuai’ region, China.

## Introduction

The both sides along the Huai River in Anhui Province, China are named as ‘Yanhuai’, which is a large, low-lying Plain located adjacent to the river and some of its tributaries. This region is an important commodity grain supplier, both regionally and nationally. The cultivation system is dominated by an annual relay double cropping system, including wheat-rice, wheat-maize and wheat-soybean. The winter wheat in this area is with a relatively fixed growing window i.e. from mid-October to early June, next year (Wang et al., 2011). The ‘Yanhuai’ area is located at the southern part of the Huaibei Plain and to the northern part of the middle and lower reaches of the Yangtze River, which is classified as a semi-arid and semi-humid area. The spatiotemporal distribution of precipitation resources within crop growing season is quite uneven due to monsoonal climate. In particular, great variability in intensity and frequency of precipitation often causes frequent waterlogging and drought, resulting in huge risk for wheat cropping.

Spatiotemporal variation in precipitation across Anhui Province has been reported (Ding et al., 2016; Jiang and Sun, 2012; Du et al., 2021). A study from over 50-year data suggests an increasing trend in annual precipitation of Anhui Province with a highly interdecadal variability in the summer rainfall. In space, precipitation in the whole Province shows a decreasing trend from Southeast to Northwest (Nie et al., 2017). Spatiotemporal distribution analysis for different magnitudes of precipitation in the ‘Yanhuai’ region suggests that the precipitation mainly occurred in the form of middle, large, and torrential rain events (Zhu et al., 2019). Uncertain and erratic distribution of precipitation is the major limitation to crop growth in the ‘Yanhuai’ basin (Nie et al., 2019). The distribution of precipitation among various growth stages of winter wheat crop is uneven, which may compromise the potential of crop yield (Yao et al., 2017). Hence, it is necessary to characterise the features of precipitation with regards to winter wheat season in the ‘Yanhuai’ region, China.

Previous studies are primarily focused on the spatiotemporal distribution of precipitation (Ye and Li, 2017; Yu et al., 2017; Sun et al., 2019) and characteristics of precipitation intensity in this region (Liu et al., 2021; Yin et al., 2021). However, the pattern of precipitation with regards to wheat growth and development has seldom been reported. As such, the existing information is inadequate in advising wheat sustainable farming under this particular climate scenario. Therefore, the objective of this study is to investigate the spatiotemporal distribution of precipitation in relating to wheat developmental stages, and quantify the characteristics of the precipitation amount and days, distribution of different grades, and continuous rain events based on climate data from 1999 to 2020 at six meteorological stations.

## Materials and methods

### Study area

**Figure 1:**
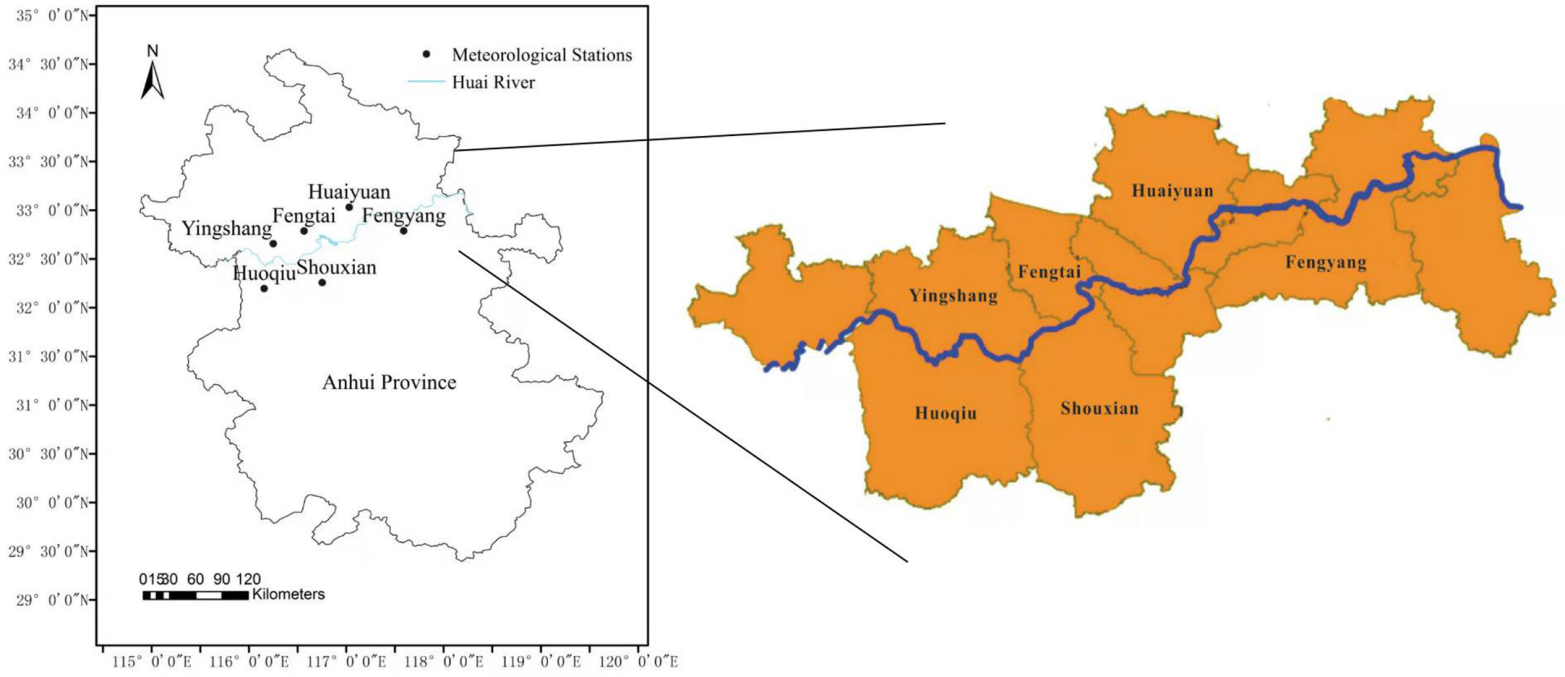
Locations of the six meteorological stations i.e. Yingshang, Huoqiu, Fengtai, Shouxian, Huaiyuan, and Fengyang in ‘Yanhuai’ region.

The Huai River runs through from West to East in North Anhui Province, China, which is located in Anhui Province with a total length of 430 kilometers. It has a drainage area of 21,434 km^2^, accounting for 15.3% of the Anhui provincial territory. Six meteorological stations i.e. Yingshang, Huoqiu, Fengtai, Shouxian, Huaiyuan, and Fengyang along the ‘Yanhuai’ in Anhui Province (**Figure 1**), located at the latitude of 32° 22’–32 °59’N and the longitude of 116°14’–117°33’E (**Table 1**), were chosen in this study. The ‘Yanhuai’ region is located at the transition zone between the warm temperate zone and subtropical zone, which has many low-lying, flood diversion and retention areas. The majority of precipitation occurs from May to October, with an annual average of 846.5 mm (Yu et al., 2007).

**Table 1:** Basic information of the six meteorological stations i.e. Yingshang, Huoqiu, Fengtai, Shouxian, Huaiyuan, and Fengyang in ‘Yanhuai’ region.

Station Name	Latitude	Longitude	Altitude (m)
Yingshang	32.39	116.14	25.2
Huoqiu	32.22	116.18	27.9
Fengtai	32.43	116.46	23.0
Shouxian	32.26	116.47	25.7
Huaiyuan	32.59	117.04	24.5
Fengyang	32.51	117.33	24.6

#### Growth stages of winter wheat

Based on local practice, the life cycle of winter wheat along the Huai River in Anhui Province was divided into seven growth stages (**Table 2**).

**Table 2:** Growth stages of winter wheat and associated calendar in Huaibei Plain, Anhui Province, China.

Growth stage	S1	S2	S3	S4	S5	S6	S7
	Sowing	Pre-winter Seedling	Overwintering	Jointing	Booting	Heading-Filling	Maturation
Time	Mid to late Oct	Nov to Dec	Jan to Feb	Mar	Early to mid-Apr	Late Apr to mid-May	Late May to early Jun

### Data source and analysis

The meteorological data obtained from the Anhui Provincial Meteorological Information Center, including daily precipitation, precipitation days, and sunlit hours, during the wheat growing seasons from 1999 to 2020 at the six meteorological stations (**Figure 1**), were obtained. These data were analyzed for characterising the precipitation distribution during wheat growing season in ‘Yanhuai’ region.

#### Grade of precipitation

According to the “Grade of precipitation standard” National GB/T28592-2012 (AQSIQ, 2012), precipitation can be divided into seven grades, based on the amount of precipitation over a 24-hour period (**Table 3**). If daily precipitation including fog, dew, and frost is ≥ 0.1 mm, it is counted as a precipitation day.

**Table 3:** Grade of precipitation over a 24-hour period.

	Grade	24-hour precipitation (mm)
G0	Scattered rain	<0.1
G1	Light rain	0.1–9.9
G2	Moderate rain	10.0–24.9
G3	Heavy rain	25.0–49.9
G4	Rainstorm	50.0–99.9
G5	Torrential rain	100.0–249.9
G6	Extraordinary rainstorm	≥250.0

#### Analysis of continuous rain features

When precipitation occurs for 3 consecutive days or more (daily precipitation ≥ 0.1 mm), it is regarded as continuous rain. A break in precipitation for 1 day is allowed in the middle of continuous rainfall events of more than 3 days, but the sunlit time of that day is considered to be less than 2 h. During continuous rain, a trace of precipitation is allowed, but the sunlit time on that day is considered to be less than 4 h (Anhui Meteorological Bureau Data Room, 1983). Based on the difference in the number of rainy days, continuous rainy days are divided into 3–6 days and ≥ 7 days (Chen et al., 2009; Liu et al., 2012).

### Data analysis

Microsoft Office Excel (Microsoft Excel 2016, Microsoft, USA) was used to analyze the data of winter wheat during each specific growth stage. Coefficient of variation (CV) was used to indicate the fluctuation of historical data.

## Results

### Distribution of precipitation during wheat growing season

Precipitation characteristics from 1999 to 2020 at six sites (**Figure 1**) are shown in **Table 4**. Precipitation during wheat growing season varied with years, with an average of six stations ranging from 141.4 to 709.2 mm over the 22 years. The highest precipitation occurred in 2017 at Huoqiu and other five stations in 2016; the lowest precipitation in all six meteorological stations was observed in 2010. The maximum precipitation was 808.5 mm (Huoqiu), while the minimum precipitation was 127.0 mm (Shouxian), and the range is 681.5 mm. The overall CV ranged from 0.27 to 0.34, indicating that variability of precipitation over wheat season was moderate.

**Table 4:** Precipitation characteristics during wheat growing season from 1999 to 2020 at six meteorological stations.

	Maximum (mm)	Minimum (mm)	Mean (mm)	CV
Yingshang	771.2	137.8	425.0	0.31
Huoqiu	808.5	166.6	455.5	0.29
Fengtai	706.3	134.1	414.0	0.28
Shouxian	780.8	127.0	416.3	0.34
Huaiyuan	589.1	153.4	374.7	0.27
Fengyang	708.3	129.5	401.1	0.32

### Distribution of precipitation during different wheat growth stages

**Figure 2:**
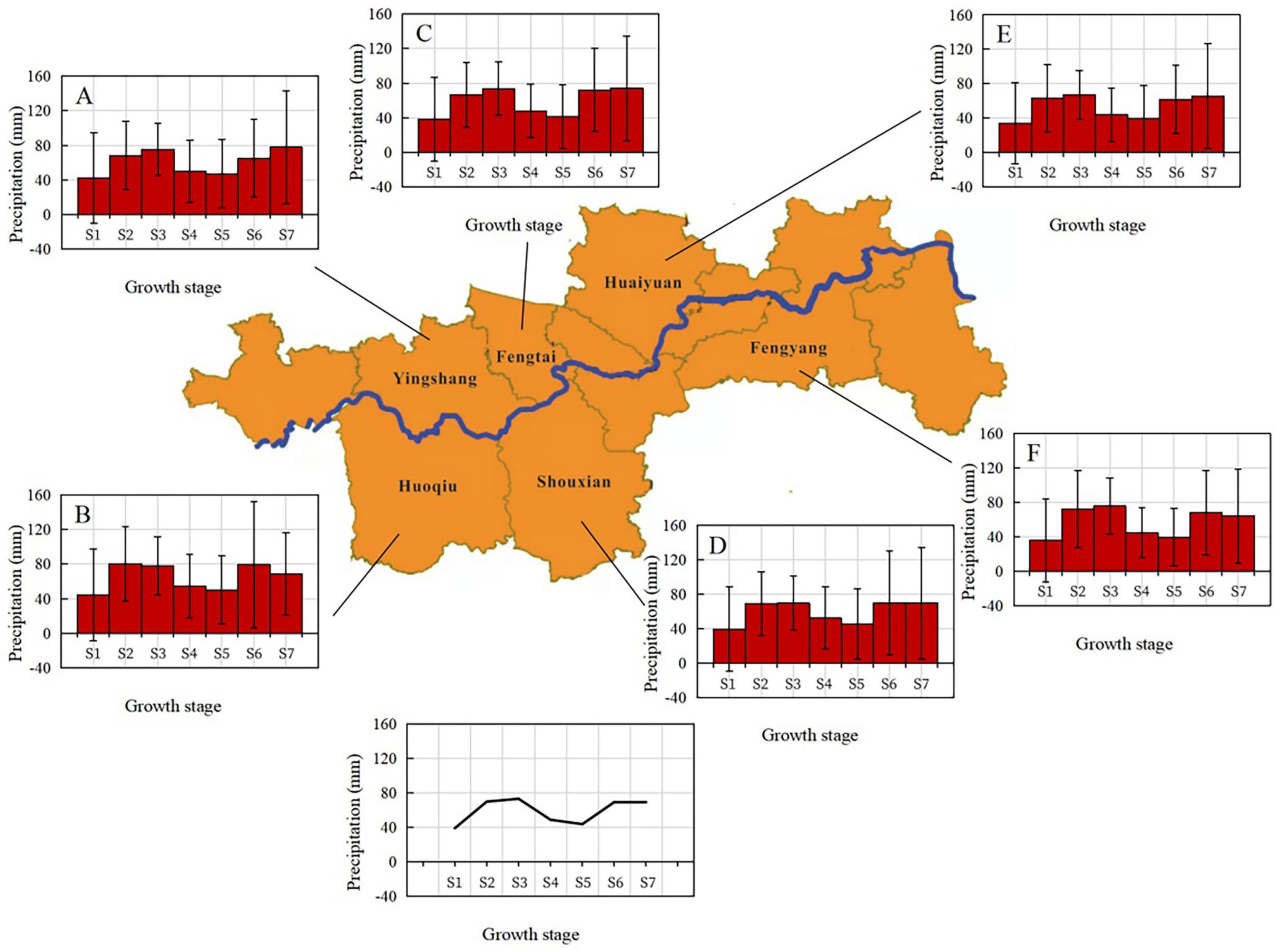
Average precipitation during wheat growth stages in ‘Yanhuai’ region i.e. **(A)** Yingshang, **(B)** Huoqiu, **(C)** Fengtai, **(D)** Shouxian, **(E)** Huaiyuan, and **(F)** Fengyang from 1999 to 2020.

The precipitation distribution during different wheat growth stages was fluctuant, and the variability for each meteorological station was similar (**Figure 2**). The average precipitation during the pre-winter seedling (S2), overwintering (S3), heading-filling (S6), and maturation stages (S7) were relatively large with a value over 60 mm; during the sowing (S1), jointing (S4), and booting stages (S5) were relatively small. The precipitation fluctuated greatly during the maturation (S7) and sowing stages (S1) but remained relatively stable during the overwintering stage (S3).

**Figure 3:**
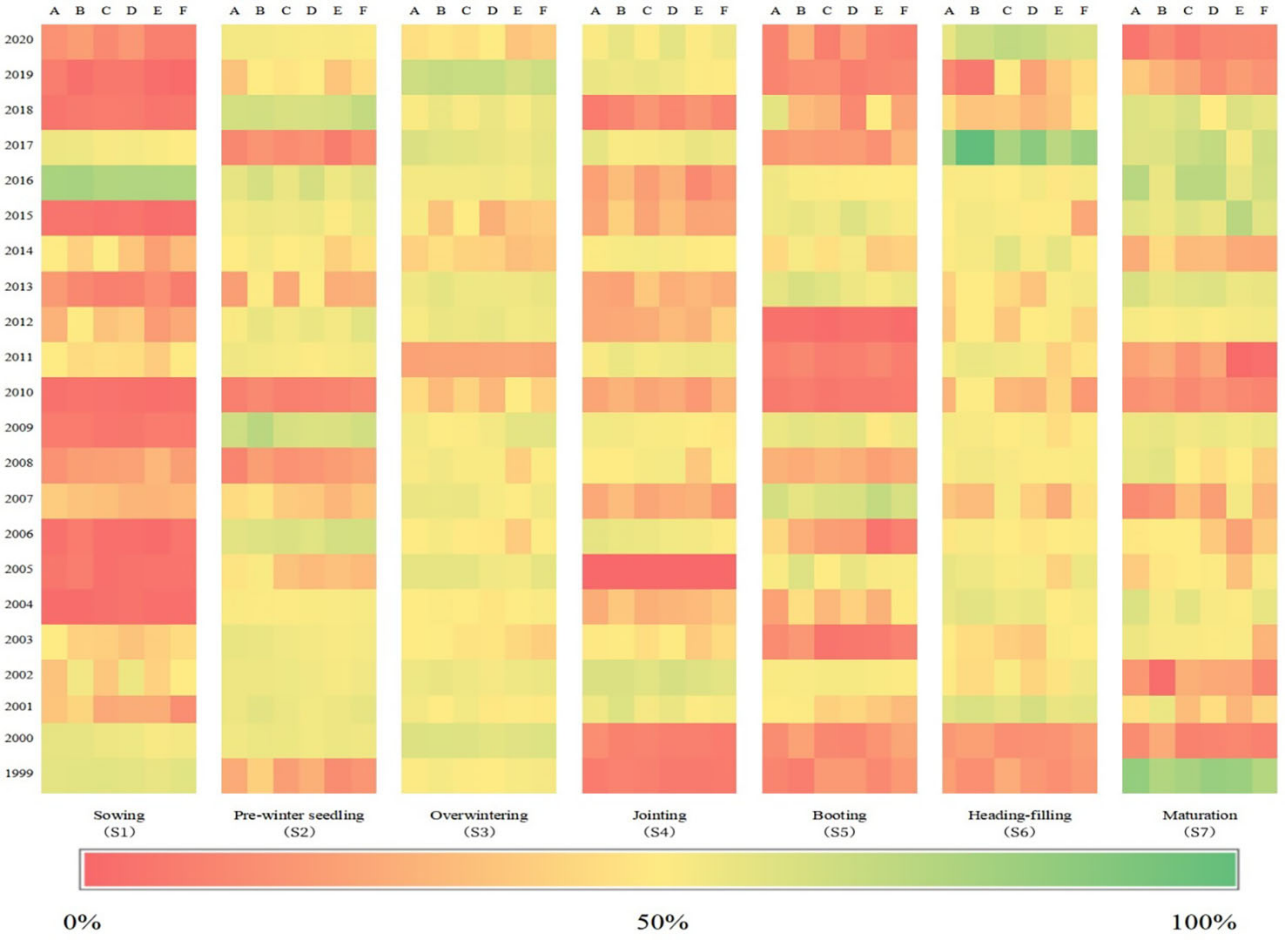
Precipitation during various wheat growth stages including Sowing (S1), Pre-winter seedling (S2), Overwintering (S3), Jointing (S4), Booting (S5), Head-filling (S6) and Maturation (S7) in ‘Yanhuai’ region i.e. **(A)** Yingshang, **(B)** Huoqiu, **(C)** Fengtai, **(D)** Shouxian, **(E)** Huaiyuan, and **(F)** Fengyang from 1999 to 2020. The greater greenness indicates more precipitation and wetter, while the greater redness indicates less precipitation and drier.

Precipitation amount distribution during seven wheat growth stages across the 22 years is presented in the heatmap (**Figure 3**). Where, 0% indicates no precipitation, and 100% represents the proportion of the current year’s precipitation to the maximum precipitation from 1999 to 2020 at this stage. The ‘Yanhuai’ region is subjected to alternation of waterlogging and drought, in addition, the frequencies of waterlogging were relatively high during the pre-winter seedling, overwintering and heading-filling stages.

Drought that persists for years was observed during the sowing and booting stage. The minimum value for the precipitation and the number of precipitation days of the six stations all appeared in 2010. Both were the fewest since 1961. The continuous lack of precipitation led to severe drought in autumn, winter and spring. During the sowing stage for wheat in 2016, the soil was so wet after more than 12 consecutive days of rain that the wheat could not be planted as scheduled. The number of days for precipitation were significantly greater than usual, while the sunshine duration was significantly lesser. In the early and middle of May in 2018, there was a lot of precipitation, especially concentrated heavy precipitation, resulting in extreme wet soil, for winter wheat in the head-filling stage. Serious waterlogging occurred during the maturation stage for wheat in 2000. Such extreme weather processes can be seen in **Figure 3**.

### Distribution of precipitation days during wheat growing season

Distribution of annual precipitation days from 1999 to 2020 at six sites i.e., Yingshang, Huoqiu, Fengtai, Shouxian, Huaiyuan, and Fengyang is shown in **Table 5**. Precipitation days of wheat growing season varied across the years, and the average precipitation days of six stations ranged from 38 days to 78 days over the 22 years. The year with the maximum precipitation days during wheat season greatly differed, while the minimum precipitation days occurred in 2010. The CV of precipitation days ranged from 0.16 to 0.17, and the randomicity of growing season precipitation days was determined as the mild variability.

**Table 5:** Annual precipitation days with regards to the whole wheat growing season from 1999 to 2020 at six meteorological stations i.e. Yingshang, Huoqiu, Fengtai, Shouxian, Huaiyuan, and Fengyang, located in Yanhuai region.

	Maximum	Minimum	Mean	CV
Yingshang	78	35	63	0.16
Huoqiu	85	41	67	0.17
Fengtai	81	37	64	0.17
Shouxian	77	36	63	0.17
Huaiyuan	72	35	60	0.17
Fengyang	78	43	62	0.16

### Different grades of precipitation during various wheat growth stages

#### Frequency in Precipitation days in different precipitation grades

**Figure 4:**
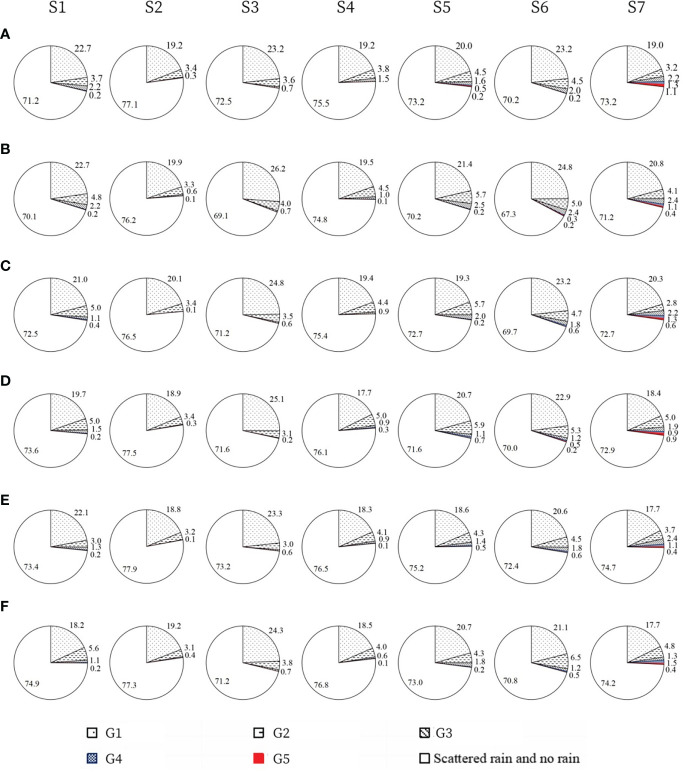
Frequencies of precipitation days in precipitation grades during wheat growth stages in Yanhuai region i.e. **(A)** Yingshang, **(B)** Huoqiu, **(C)** Fengtai, **(D)** Shouxian, **(E)** Huaiyuan, and **(F)** Fengyang from 1999 to 2020.

The frequencies of precipitation days in different grades during various wheat growth stages across six sites i.e., Yingshang, Huoqiu, Fengtai, Shouxian, Huaiyuan, and Fengyang are shown in **Figure 4**. Light rain, moderate rain, and heavy rain occurred during seven wheat growth stages. During each wheat growth stage, for the different-grade rain, the light rain occurs the most frequently, followed by the moderate rain, and the heavy rain and rainstorm occur less frequently than the moderate rain, while torrential rain is rare. Rainstorm occurred during the sowing (0.2-0.4%), booting (0.2-0.7%), heading-filling (0.2-0.6%), and maturation (0.9-1.5%) stages but did not occur during the overwintering stage. Torrential rain only occurred during the heading-filling (0-0.2%) and maturation (0.4-1.1%) stages. It is noted that extraordinary rainstorms were not observed in none of the six meteorological stations.

#### Precipitation percentage in different precipitation grades

**Figure 5:**
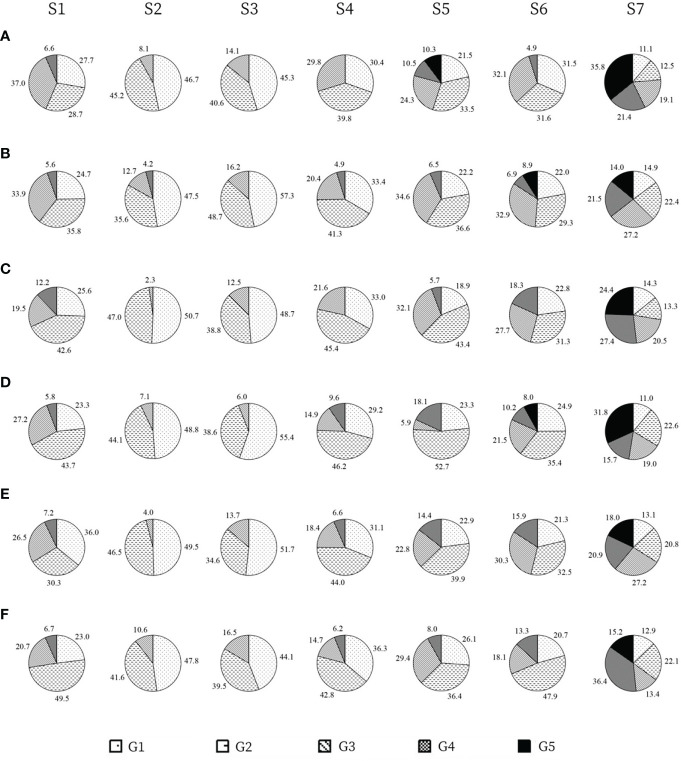
Precipitation amount percentage at different precipitation grades during wheat growth stages in ‘Yanhuai’ region i.e. **(A)** Yingshang, **(B)** Huoqiu, **(C)** Fengtai, **(D)** Shouxian, **(E)** Huaiyuan, and **(F)** Fengyang from 1999 to 2020.

The percentages of precipitation from various grades at different wheat growth stages at six sites i.e. Yingshang, Huoqiu, Fengtai, Shouxian, Huaiyuan, and Fengyang are shown in **Figure 5**. The percentages of rainfall in different grades and a significant difference during seven wheat growth stages is shown (**Figure 5**). Light rain and moderate rain were dominant during the pre-winter seedling and overwintering stages, while light, moderate, and heavy rain were dominant during the sowing, jointing, and booting stages. Torrential rain only occurred during the maturation stage at six sites.

### Distribution of continuous rain during different wheat growth stages

#### Frequency of continuous rain during different wheat growth stages

**Figure 6:**
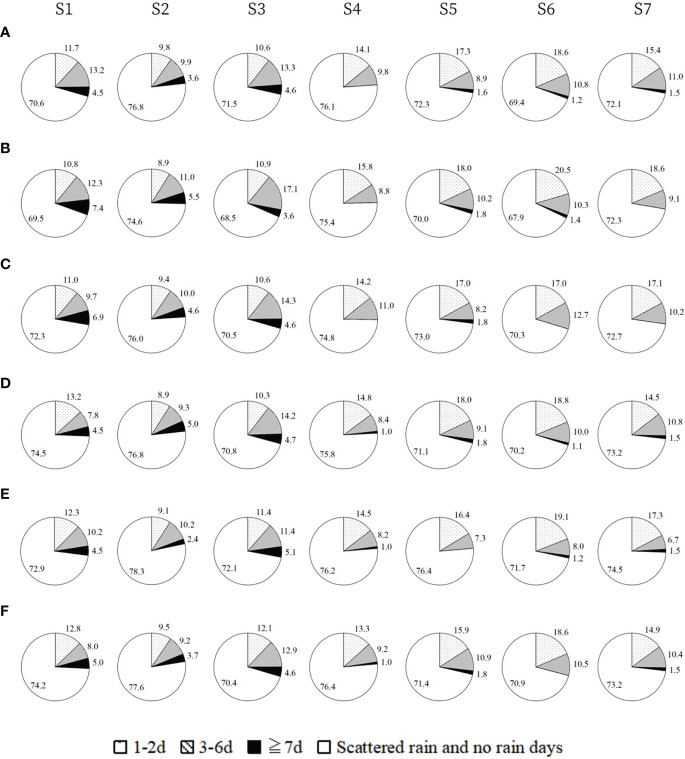
Frequencies of continuous rain during different winter wheat growth stages in Yanhuai region i.e. **(A)** Yingshang, **(B)** Huoqiu, **(C)** Fengtai, **(D)** Shouxian, **(E)** Huaiyuan, and **(F)** Fengyang from 1999 to 2020.

The frequencies of continuous rain during each wheat growth stage at six sites i.e. Yingshang, Huoqiu, Fengtai, Shouxian, Huaiyuan, and Fengyang are shown in **Figure 6**. The frequencies of continuous rain varied among different growth stages. Continuous rain lasting more than 7 days occurred more frequently during the sowing (4.5-7.4%), overwintering (2.4-5.5%), pre-winter seedling (3.6-5.1%) stages, and rarely occurred during the other growth stages. It can be seen from the figure that continuous rain is more likely to occur during the early growth stages of winter wheat. The longest continuous rainy days occurred during the sowing stage were 12 days (Yingshang, Huoqiu, Shouxian, and Huaiyuan) and 14 days (Fengtai and Fengyang), respectively.

#### Precipitation percentage of continuous rain during different wheat growth stages

**Figure 7:**
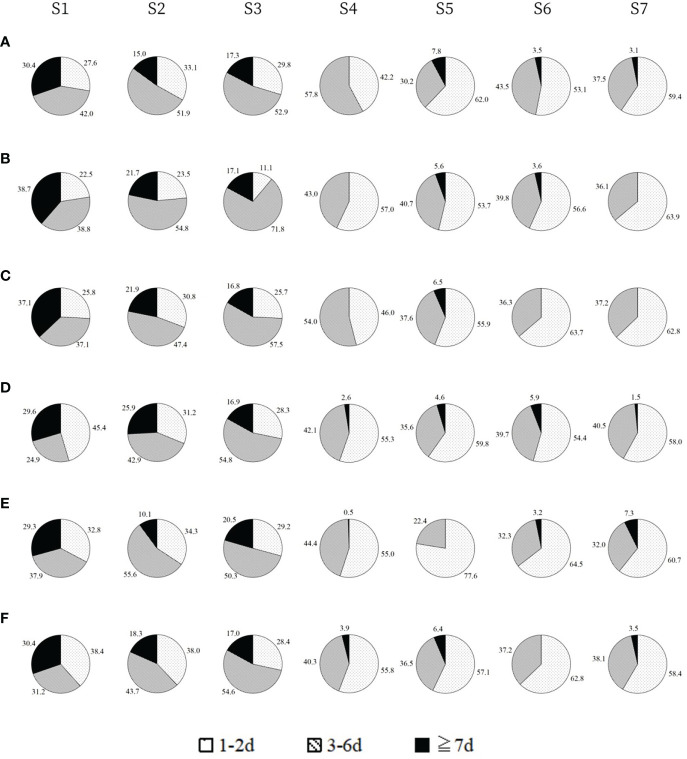
Precipitation amount percentages of continuous rain during different wheat growth stages in Yanhuai region i.e. **(A)** Yingshang, **(B)** Huoqiu, **(C)** Fengtai, **(D)** Shouxian, **(E)** Huaiyuan, and **(F)** Fengyang from 1999 to 2020.

The precipitation percentage of continuous rain during each growth stage at six sites i.e. Yingshang, Huoqiu, Fengtai, Shouxian, Huaiyuan, and Fengyang is shown (**Figure 7**). The precipitation percentage of continuous rain lasting 3–6 days was highest during the overwintering stage (50.3-71.8%), followed by the pre-winter seedling stage (42.9-55.6%). The precipitation percentage of continuous rain lasting more than 7 days was highest during the sowing stage (29.3-38.7%), followed by the pre-winter seedling (10.1-25.9%) and overwintering (16.8-20.5%) stages. The data reveals that the precipitation type during the early growth stages (S1-S3) is mainly dominated by continuous rain. The maximum precipitation occurred during the sowing stage (Huoqiu and Fengyang) and the maturation stage (Yingshang, Fengtai, Shouxian, and Huaiyuan).

## Discussion

### Characteristics of waterlogging and drought

The climatic productivity potential of winter wheat in the Huai River Basin is restricted by precipitation episode in the growing season (Yuan et al., 2016). Precipitation appears sufficient during the growing season of winter wheat in the region, nevertheless the distribution of precipitation is fairly uneven, causing high risk of drought or waterlogging events. The impacts are also influenced by meteorological condition e.g. sunshine, soil environment e.g. soil texture and crops themselves e.g. water demand, root characteristics (Dickin and Wright, 2008; Licausi and Perata, 2009; Katerji et al., 2010; Zhang et al., 2010; Araki et al., 2012b; Zhang et al., 2021). This study mainly considered the influence of precipitation and sunshine, and thus focused on characterising the precipitation during winter wheat growing season.

The representative soil types in ‘Yanhuai’ region, include sand ginger black soil, tidal soil and silty soil. The soil is short of organic matter and hard to keep soil water. Root growth has a close relationship with soil water content (Lv et al., 2010). Either too much or insufficient soil water are adverse conditions for root growth. The soil moisture refers to the soil water status in the main root system activity layer of crops (Inoue et al., 2004). In this study, soil moisture was used as an index to determine whether the soil is too dry or too wet. And soil moisture measured is normally at the depth of 0-20 cm, 20-40cm in the particular region due to shallow soil layers. Soil moisture less than 60% is considered drought, while more than 80% is waterlogging. Therefore, in the growing season of wheat, the influence of soil moisture profile and soil texture on available soil water holding capacity and wheat root will be considered in future. Due to the differences in drainage and irrigation conditions and the cost, many farmers did not take irrigation measures, so irrigation and drainage methods were not investigated in this study. The losses caused by drought and waterlogging with the same intensity to areas with good drainage and irrigation conditions are less than those with poor conditions, which is the manifestation of regional vulnerability differences (Zhang et al., 2007). Drought and flood indicators should take more account of the impact on agriculture. On the basis of collecting crop growth information of wheat growing season, combined with some meteorological, phenological and soil observation data, the disaster monitoring, diagnosis and analysis model will be established (Ge et al., 2016; Ge et al., 2017).

The water requirement of winter wheat of the whole growing period and different growth stages during 1961-2010 in China were estimated (Sun et al., 2013). Combined with the spatial distribution characteristics of precipitation, the water-meeting situation of winter wheat during the growing season was analyzed. Results revealed that water requirement of winter wheat in Anhui Province is 300-400 mm. By comparing the water deficit of the most serious areas, the growth stage of the water deficit of the least seriously is from sowing to over-wintering and the growth stage of the water deficit of the most severe is from flowering to ripening. However, the results of this study showed that winter wheat was always dry during sowing stage and waterlogging occasionally occurred during heading-filling stage. The metrological data from the 22 years indicated that ‘Yanhuai’ region was subjected to alternation of waterlogging and drought. The frequencies of waterlogging were relatively high during the pre-winter seedling, overwintering and heading-filling stages. Drought that persists for years was observed during the sowing and booting stage. The continuous lack of precipitation led to severe autumn, winter and spring drought in 2010. Waterlogging and drought that persists for years or the alternating occurrence of waterlogging and drought (from drought to waterlogging and from waterlogging to drought) in the same year are often observed.

### Cropping risk and cultivation practices of wheat production in ‘Yanhuai’ region

The ‘Yanhuai’ region is an important commodity grain production base. Good knowledge of waterlogging and drought behaviors is of great importance in the planning and management of agricultural activities in this region. The interannual fluctuation of precipitation is large, and the interannual relative variability of precipitation is the largest in autumn. Maintaining the balance of groundwater exploitation and replenishment, combining drainage and storage, aiming at improving the efficiency of water resources utilization, vigorously developing efficient water-saving irrigation technology is the key measure to deal with the adverse effects of frequent meteorological droughts on crop production, and also an important measure to ensure water resources and food security.

As observed in this study, continuous rains may occur at each stage in winter wheat growing season. In the mechanized agriculture, these continuous rains may delay crop sowing and impact the harvesting activities (Chen et al., 2009). Further, frequent rains and low temperature during the pre-winter seedling and overwintering stages, and sudden temperature increases during jointing stage, could cause severe aphid outbreaks. After heading and flowering, aphids will erupt again in high temperature and humidity (Zhao et al., 2016). The booting stage is the sensitive stage of wheat waterlogging injury (Li et al., 2001). The number of rainy days during the heading-filling stage is the most important factor for the occurrence of scab (Zhao et al., 2016). Waterlogging during booting to maturation stage which are the key periods of yield formation can lead to reduced production (Li et al., 2001). Continuous rain during maturation stage, may induce pre-harvest sprouting in winter wheat (Zhao et al., 2016). To minimize this damage, it is necessary to plow the soil on sunny days after rice harvest. After rainfall in spring, timely dredge furrows can ensure the smooth drainage of the field, to prevent waterlogging, and reduce disease, insect and grass damage. Selecting suitable fertilizer and growth regulator during different growth stages can alleviate waterlogging damage to a certain extent (Gao et al., 2020). Considering the water and heat resources and the actual situation of agriculture, it is of great significance to modify the tillage system in minimise impacts of likely waterlogging and drought.

## Conclusion

Precipitation during wheat growing season varied greatly with years in the wheat producing regions of Huai river in Anhui Province, China. Precipitation is unevenly distributed across winter wheat developmental phases. Continuous rain occurred frequently during the pre-winter seedling and overwintering stages, and timely drainage measure is needed at this time. There is less precipitation in key water demand periods such as jointing and booting, so irrigation should be timely. The precipitation fluctuated greatly in sowing, heading, filling and maturity, and drought and flood occurred from time to time. Our work guides to take effective measures for irrigation or drainage management based on the precipitation characteristics of winter wheat at different growth stages. Our findings provide a scientific basis for disaster prevention and mitigation in wheat sustainable production in the ‘Yanhuai’ region or similar climate zones.

## Data availability statement

The original contributions presented in the study are included in the article/supplementary material. Further inquiries can be directed to the corresponding authors.

## Author contributions

DF did data collection and analysis, as well as drafted and finalized the manuscript; JH contributed to data analysis and the manuscript drafting; WS contributed to data analysis and figure presentation; NU contributed to manuscript revision; SJ contributed to data collection and analysis, and manuscript revision. YS supervised the work and finalized the writing. All authors contributed to the article and approved the submitted version.
